# PROTOCOL: Mobile apps to reduce depressive symptoms and alcohol use in youth: A systematic review and meta‐analysis

**DOI:** 10.1002/cl2.1222

**Published:** 2022-03-14

**Authors:** Olivia Magwood, Ammar Saad, Dominique Ranger, Kate Volpini, Franklin Rukikamirera, Rinila Haridas, Shahab Sayfi, Jeremie Alexander, Yvonne Tan, Jennifer Petkovic, Kevin Pottie

**Affiliations:** ^1^ C.T. Lamont Primary Health Care Research Centre Bruyère Research Institute Ottawa Ontario Canada; ^2^ Interdisciplinary School of Health Sciences, Faculty of Health Sciences University of Ottawa Ottawa Ontario Canada; ^3^ School of Epidemiology and Public Health University of Ottawa Ottawa Ontario Canada; ^4^ Children's Hospital of Eastern Ontario Research Institute Ottawa Ontario Canada; ^5^ Faculty of Medicine University of Ottawa Ottawa Ontario Canada; ^6^ Faculty of Science University of Ottawa Ottawa Ontario Canada; ^7^ Department of Biology, Faculty of Science University of Ottawa Ottawa Ontario Canada; ^8^ Department of Biology, Faculty of Arts and Science Queen's University Kingston Ontario Canada; ^9^ Department of Biomedical and Molecular Sciences, Faculty of Arts and Science Queen's University Kingston Ontario Canada; ^10^ Bruyère Research Institute University of Ottawa Ottawa Ontario Canada; ^11^ Department of Family Medicine, Faculty of Medicine University of Ottawa Ottawa Ontario Canada; ^12^ Department of Family Medicine, Schulich School of Medicine & Dentistry Western University London Ontario Canada

## Abstract

**Background:**

Depressive symptoms and alcohol use in youth doubled in the first year of the COVID‐19 pandemic. The COVID‐19 pandemic has created sustained disruption in society, schools, and universities, including increasing poverty and discrimination. Public health restrictions have caused isolation and reduced social and emotional support. Together, these factors make depressive symptoms and alcohol use in youth a global public health emergency. Mobile applications (apps) have emerged as potentially scalable intervention to reduce depressive symptoms and alcohol use in youth that could meet increased demands for mental health resources. Mobile apps may potentially reduce psychological distress with accessible technology‐based mental health resources.

**Objectives:**

This systematic review and meta‐analysis aims to assess the effect of mobile apps on depressive symptoms and alcohol use in youth.

**Search Methods:**

We will develop a systematic search strategy in collaboration with an experienced librarian. We will search a series of databases (MEDLINE, Embase, PsycINFO, CINAHL, CENTRAL) from January 2008 to July 2021.

**Selection Criteria:**

Following the PRISMA reporting guidelines for systematic reviews, two independent reviewers will identify eligible studies: randomized controlled trials on mobile apps for the management of depressive disorders (depression and anxiety) and alcohol use in youth aged 15–24 years of age.

**Data Collection and Analysis:**

Eligible studies will be assessed for risk of bias, and outcomes pooled, when appropriate, for meta‐analysis. Heterogeneity, if present, will be examined for gender. ethnicity, and socioeconomic status contributions. A narrative synthesis will highlight similarities and differences between the included studies. We will report GRADE summary of finding tables.

## 1 BACKGROUND

### 1.1 The problem, condition, or issue

The COVID‐19 pandemic has had a grave effect on depressive symptoms and alcohol use in adolescents and youth. Depressive symptoms in youth have doubled in the first year of the COVID‐19 pandemic and pooled estimates suggest that one in four youth globally are experiencing clinically elevated depression symptoms and that an increase in mental health care utilization is expected (Racine et al., 2021).

Heavy use of alcohol has increased 43% in youth, comparing 2012 with 2020 data (Pollard et al., 2020). The COVID‐19 pandemic has created sustained disruption in society, schools, and universities, including increasing poverty and discrimination and public health restrictions have caused isolation and reduced social and emotional support (Glover et al., 2020). Together, these factors make depressive symptoms in youth a global public health emergency (Racine et al., 2021).

The World Health Organization estimates that 10% of youth worldwide experience mental health disorders (World Health Organization [WHO], 2018). Yet despite this high prevalence, youth with mental health and/or substance use disorders rarely access mental health or primary care services due to personal and systemic barriers (Ross & Connors, 2018). The social stigma of asking for help, limitations in community services, and lack of awareness amongst primary care providers, teachers, and parents often leave the youth alone and untreated (Cash et al., 2018; Malla et al., 2019). Post‐secondary students are also faced with greater stress from increased academic demands, adjusting to a new environment, and developing a new support system. Under such conditions, depressive disorder diagnoses are common among students, with one in four students treated for a mental disorder, one in five reporting thoughts about suicide, 9% reporting having attempted suicide and nearly 20% reporting self‐injury (Liu et al., 2019). Failure to receive proper mental healthcare has costly consequences for youth, including depression, suicide, expulsion in early education, later unemployment, and higher incarceration rates (Cash et al., 2018). Furthermore, not only do depressive disorders and alcohol use disorders often persist into adulthood, but these disorders are reported to intensify in severity and become more challenging to treat with age (Pedrelli et al., 2015). The COVID‐19 pandemic and related public health restrictions have had a grave effect on youth and addressing the mental health of youth is a now a public health priority.

Given the stigma associated with accessing mental health services and the universality of mobile smartphones, especially among youth, mobile health applications (apps) offer a unique opportunity to provide services in a non‐judgemental, readily accessible, and large‐scale manner. With greater than three billion smartphone users worldwide and more than 165,000 health‐related apps available for mobile download (Terry, 2015), applying a mobile app approach to mental health services may offer a ubiquitous means to help manage the current healthcare demand. Moreover, youth are particularly well positioned to benefit from digital health resources given their increasing acceptance and usage of technology (Hyden & Cohall, 2011). Mobile apps may also offer a solution to youth who would otherwise lack the independence to access services targeting the prevention and management of mental health disorders and stress on their own or would prefer the discretion and anonymity that digital platforms can offer (Grist et al., 2017). Physician prescribing of mobile apps further endorses digital interventions as an essential resource to engage youth in the prevention and self‐management of mental health issues. Indeed, examining the effectiveness of digital mental health interventions are recognized research priorities (Hollis et al., 2018).

Gender and socioeconomic status disparities exist among adolescents in the receipt of in‐person treatment for major depression and anxiety (Cummings & Druss, 2011), as well as in the treatment for alcohol use disorders (Alegria et al., 2011), and these factors may lead to health inequities. As this mobile app research field matures into larger and longer term outcomes it will be important to consider equity factors in relation to effectiveness. Equity factors are frequently described according to the acronym PROGRESS‐Plus: place of residence, race/ethnicity/culture/language, occupation, gender/sex, religion, education, socioeconomic status, and social capital. The term “Plus” refers to personal characteristics (i.e., age, disabilities), relationship features (i.e., exclusion from school, parent drug use), and time‐dependent relationships (i.e., leaving the hospital or other times when an individual might be temporarily disadvantaged) (O'Neill et al., 2014).

While research has focused on evaluating the functionality of mobile apps, less is known about the effectiveness to reduce distress in students and youth, in particular. We believe it is timely to evaluate the effectiveness of mobile apps for depressive disorders and alcohol use in youth.

### 1.2 Description of the condition

The COVID‐19 pandemic has had a grave effect on depressive symptoms and alcohol use in adolescents and youth. Depressive symptoms in youth have doubled in the first year of the COVID‐19 pandemic and pooled estimates suggest that one in four youth globally are experiencing clinically elevated depression symptoms and that an increase in mental health care utilization is expected (Racine et al., 2021).

Heavy use of alcohol has increased 43% in youth, comparing 2012 with 2020 data (Pollard et al., 2020). The COVID‐19 pandemic has created sustained disruption in society, schools, and universities, including increasing poverty and discrimination and public health restrictions have caused isolation and reduced social and emotional support (Glover et al., 2020). Together, these factors make depressive symptoms in youth a global public health emergency (Racine et al., 2021).

### 1.3 Description of the intervention

**Figure 1:**
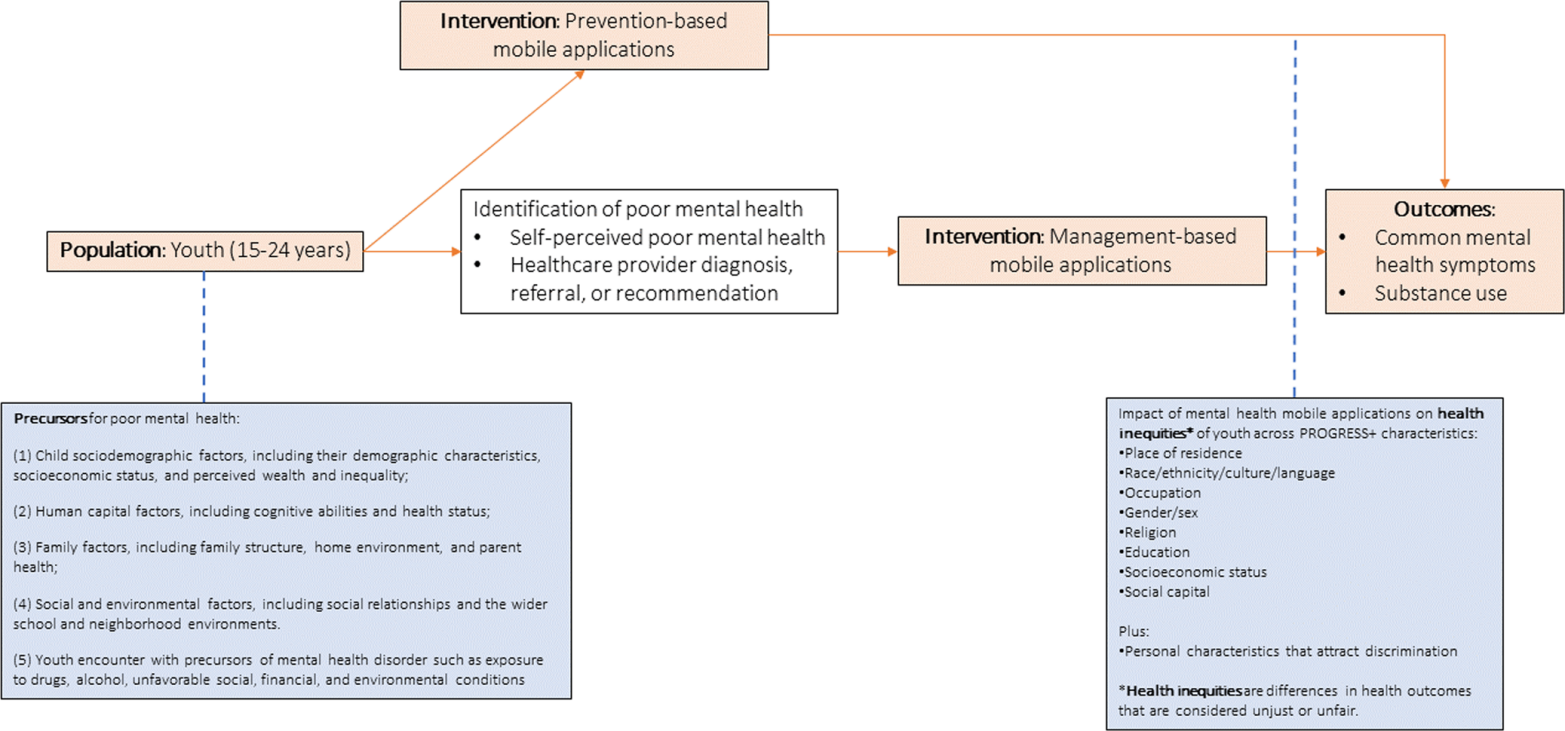
Logic model for mobile applications for youth mental health

This systematic review will focus on mobile applications (apps) that target the management of mental health disorders and psychological stress, hereafter referred to as “mental health mobile apps” (see Figure 1). Mental health mobile apps are health apps available on a mobile device (smartphone, tablet, or phablet), which can be used by both patients and their health care providers separately. Mental health apps vary in design and functionality but share the commonality of targeting either the prevention or management of a broad range of mental health disorders (e.g., depression, anxiety, PTSD), as well as elevated psychological stress levels as the root cause of mental health morbidity. We define management‐based mobile apps as interventions that are used to manage mental health disorders when they exist and/or control the exacerbation or severity of their symptomatology once they occur. These apps are intended to serve as platforms that deliver mental health services accessed by youth at any time or place in the absence of a direct interaction with the healthcare provider or specialist.

The U.S. National Institute of Mental Health has identified five areas of mental health app development, based on functionality, of relevance to youth with mental health concerns (U.S. Department of Health and Human Services et al., 2019):

1. Self‐management apps: “Self‐management” means that the user puts information into the app so that the app can provide feedback. For example, the user might set up medication reminders, or use the app to develop tools for managing stress, anxiety, or sleep problems. The *Optimism* app is an example of a symptom tracking app for mood disorders that asks users to fill in information every day about their symptoms and notable events. This information is then compiled into several charts and graphs intended to make it easy to recognize patterns and identify triggers (Psyberguide, 2018a). Similarly, the *eMoods* app asks users to record their mood and anxiety daily. The user is able to generate a monthly report which they can share with their doctor to help recognize patterns in the user's daily life (PsyberGuide, 2018b).
2. Apps for improving thinking skills: Apps that help the user with cognitive remediation (improved thinking skills). These apps are often targeted toward people with serious mental illnesses, but can also benefit the general public. For example, *Elevate* aims to improve the user's ability to focus, reading comprehension, and memory with a series of quick exercises (Elevate, n.d.). Similarly, *Lumosity* provides cognitive training tasks that each target a particular core cognitive ability and are grouped into five categories by target domain: speed of processing, attention, memory, flexibility, and problem solving (Hardy et al., 2015).
3. Skill‐training apps: Skill‐training apps may feel like games as they help users learn new coping or thinking skills. For example, the user might watch an educational video about anxiety management or the importance of social support. Next, the user might pick some new strategies to try and then use the app to track how often those new skills are practiced. For example, *Headspace* is a meditation app that teaches users exercises they can perform during sudden meltdowns (Headspace Inc, 2020). Similarly, *MoodMission* asks users how they feel at a particular time and then offer “missions” which may be behavior‐based (e.g., learn how to knit, crochet, or sew), physical‐based (e.g., push ups), thought‐based (e.g., decatastrophize) or emotion‐based (e.g., breath and emotions meditation) (Psyberguide, 2018c).
4. Illness management, supported care: This type of app technology adds additional support by allowing the user to interact with another human being. The app may help the user connect with peer support or may send information to a trained health care provider who can offer guidance and therapy options. For example, the *NotOK* app was developed by youth and allows the user to reach out to five close contacts to let them know that they are not OK, and in need of peer support (Bug & Bee, 2018).
5. Passive symptom tracking: These apps collect data using the sensors built into smartphones. These sensors can record movement patterns, social interactions (such as the number of texts and phone calls), behavior at different times of the day, vocal tone and speed, and more. Such apps may be able to recognize changes in behavior patterns that signal a mood episode such as mania, depression, or psychosis before it occurs. *StudentLife* is an example of such an app that uses raw data from the phone's microphone, accelerometer, light sensor, and location sensors to find patterns in sleep, conversation, and activity data and correlates this to symptoms of depression (Clark, 2014; Wang, 2014).

Mobile health apps for healthcare are generally not distributed through healthcare providers or settings (Leigh & Flatt, 2015). Rather, mental health apps are more widely available to patients in app stores, so users are generally left to evaluate their app choices based on user ratings (Chiauzzi & Newell, 2019). Unfortunately, these ratings are based on subjective experiences of users, typically from a usability or visual standpoint, and are not reflective of an app's quality in terms of improving health outcomes (Bidargaddi et al., 2017). Additionally, it should be noted that the level of comprehensiveness of information and adherence to best‐practice guidelines do not correlate with average user mental health app ratings (Nicholas et al., 2017). Indeed, retention is highly variable even within controlled studies among mental health user (Torous et al., 2018).

### 1.4 How the intervention might work

Numerous barriers exist for youth to access appropriate mental health care resources and services (Racine et al., 2021). These includes a lack of health human resources trained to effectively deliver mental health care to youth, silos of mental health services, stigma, and inadequate availability of appropriate youth mental health care at the primary care level (Kutcher, 2012). Mobile mental health apps offer a range of resources that make therapeutic techniques more accessible and portable. They have the potential to overcome treatment barriers, such as geographic location and financial cost, and to provide effective interventions for clinical populations (Van Ameringen et al., 2017). They may serve as an attractive option for underserved populations, such as those of low socioeconomic status. Indeed, with the cost of apps being significantly less than that of traditional care, they could provide some form of care to populations where help may not be affordable or available. Apps could also alleviate the burden on the health care system by providing a self‐help option for those with mild depressive symptoms. This would reserve the limited and specialized services for more severe cases.

There are also potential benefits to using mobile apps in conjunction with usual treatment and care. Using mobile technology as a supplement to usual care may enhance the delivery of treatments (Lindhiem et al., 2015). Multipurpose or treatment apps may reduce symptoms and the need for in‐person appointments with clinicians, thereby limiting the inconvenience of geographical barriers, time or financial costs for the patient, and alleviating the workload of a clinician. The potential benefits of any mobile app, however, are contingent upon its effective role in treatment (Van Ameringen et al., 2017). Indeed, a limited body of evidence suggests that mobile interventions for suicidal ideation may be effective, but it is unclear whether these reductions would be clinically meaningful (Perry et al., 2016; Witt et al., 2017).

Whether used alone or in conjunction with usual treatment, mobile apps provide users with the opportunity to increase their awareness about their symptoms or daily habits which contribute to poor mental health. With increased awareness comes more opportunities to intervene through skill‐building activities or direct users to appropriate care or peer support aimed at improving symptoms of depression, anxiety, substance use, and sleep patterns. These mobile apps may help to promote user autonomy and independence by facilitating an increase in self‐awareness and self‐efficacy skills (Prentice & Dobson, 2014). Furthermore, there is growing evidence that developing social and emotional capabilities are important outcomes for youth (McNeil et al., 2012) which support the achievement of positive life outcomes, including educational attainment, employment, and health. Capabilities such as resilience are also increasingly cited as being the foundations of employability.

Finally, as with any health intervention or advancement in technology, there is the potential for harm. Data security for mental health apps is a widespread concern (Powell et al., 2014). Furthermore, many available mental health mobile apps target specific disorders and label their users with a diagnosis. Much research has suggested that this labeling process could be harmful and stigmatizing (Moses, 2009). Finally, there is increasing concern for digital dependency, especially among youth. The relationship between app exposure and health in adolescents may follow an “inverted U” pattern, that is, that very high exposure and very low exposure might both be associated with poorer mental health outcomes than moderate amounts of usage (Christakis, 2019).

### 1.5 Why it is important to do this review

Several published reviews have examined the early impact of mobile applications on mental health outcomes, but the heterogeneity in the definition of the intervention, classification of diseases, and selection of populations rendered a rather distorted portrait that is not necessarily relevant for youth. Two systematic reviews found promising evidence supporting the role such applications play in improving common mental health conditions such as depression, anxiety, and substance use (Donker et al., 2013; Rathbone & Prescott, 2017). However, their populations of interest encompassed participants of all ages, preventing an assessment of youth‐specific effect estimates. Conversely, three systematic reviews found limited or scarce evidence on the safety and efficacy of mobile applications in managing common mental health conditions (Firth & Torous, 2015; Grist et al., 2017; Nicholas et al., 2015). To the best of our knowledge, no reviews of literature have reported on the impact of such interventions specifically on youth. Our proposed review will synthesize knowledge of the effectiveness of youth mental health applications for depressive disorders and alcohol use disorders.

The World Health Organization has called for a comprehensive and effective response to the global burden of mental health disorders (WHO, 2013). In its 2013–2020 Mental Health Action Plan, WHO recommended the promotion of self‐help through evidence‐based digital and mobile technology (WHO, 2013). Several states such as the UK and Australia have commenced the implementation of nation‐wide strategies to prioritize the use of digital technologies in health care. We anticipate that the findings of our youth‐focused systematic review will inform policy makers, stakeholders, and government bodies about the magnitude to which mobile applications can impact mental health‐specific outcomes among youth populations. As well, we anticipate that our findings will have implications on future research in the area of mHealth and youth mental health.

## 2 OBJECTIVES

The objective of this review is to synthesize the best available evidence on the effectiveness of mobile apps for the reduction of depressive symptoms (depression and generalized anxiety) and alcohol use in youth.

## 3 METHODS

### 3.1 Criteria for considering studies for this review

#### 3.1.1 Types of studies

The methodology described below was developed according to the Preferred Reporting Items for Systematic Review and Meta‐Analysis Protocols (PRISMA‐P) and the Methodological Expectations of Campbell Collaboration Intervention Reviews (MECCIR) (Higgins et al., 2019; Moher et al., 2015; Welch et al., 2012). This protocol was registered with PROSPERO (ID# 169848).

##### 3.1.1.1 Stakeholder engagement

To ensure the relevancy of this review to our target population, we will include youth aged 15–24 as members of our review team (Kendall et al., 2017). These team members will actively contribute to the research processes, including development of this protocol and interpretation of research findings. Their input will be constructive to other team members to understand the perceived gaps in mobile applications for depressive disorder and alcohol use in youth.

##### 3.1.1.2 Types of study designs

Our review is designed to retrieve all relevant randomized controlled trails concerning use of mobile apps to reduce depressive symptoms and alcohol use for youth.

#### 3.1.2 Types of participants

This review will include adolescents and youth aged 15–24 years of age with depressive symptoms or alcohol use, related to depression and generalized anxiety. We will include studies that report on depressive symptoms, or alcohol use or anxiety but will not set a threshold for these symptoms for inclusion.

We will exclude studies that focus specifically on bipolar disorder, psychotic disorders, eating disorders, and other substance use disorders besides alcohol use. We have decided to include alcohol use within our study because alcohol is often increased with psychological distress, and alcohol is a known depressant.

We will only include studies that measure these illnesses, and we will not use a threshold. Studies will be eligible if they report on depressive symptoms or alcohol use.

This review will include youth and adolescents aged 15–24 years of age. This age range was selected to coincide with the United Nations definition of youth (UN, n.d.). We will not exclude populations on the basis of gender, socioeconomic status, geographic location or other personal characteristics. If we identify studies with participants outside of our age range (e.g., high school students aged 12–15 years or young adults aged 25–30 years) we will include the study if (1) the mean age of the study participants is between 15 and 24 years, or (2) disaggregated data is available from the authors.

#### 3.1.3 Types of interventions

This systematic review will focus on mobile applications that target the management of depressive symptoms or alcohol use. Mobile apps are health apps available on a mobile device (smartphone, tablet, or phablet), which can be used independently by the public. We will include studies that include mobile apps that are being used among youth who have (or are assumed to have) depressive symptoms or alcohol use at the time of implementation. We will exclude any platforms that merely connect patients with their healthcare provider via video‐conferencing or voice calls, such as telemedicine. We will exclude wellness and prevention only focused mobile apps as our primary outcomes will be depressive symptoms and alcohol use. The purpose of the intervention will be determined by reviewing the rationale and theory behind developing and implementing the intervention as described by authors in the primary study.

Interventions which are not eligible for inclusion are:

- Mobile phones for sending Short Message Service (SMS) messages
- Web‐based interventions
- Interventions delivered through social media platforms such as Facebook, Twitter, and Instagram
- Interventions delivered through email
- Telemedicine services that only provide direct interaction between patients and their remote healthcare provider via teleconferencing

##### 3.1.3.1 Types of comparisons

Eligible comparisons will include usual care, standard care, sham, placebo, no intervention, or wait‐list control.

#### 3.1.4 Types of outcome measures

Eligible studies must include at least one of the following primary outcomes:

Depressive symptoms
Alcohol use

We will also include the following secondary outcomes when reported in eligible studies, but these will not be used to determine eligibility for inclusion in the review:

Psychological distress symptoms
Anxiety symptoms

All outcomes must be assessed with validated mental health (depression, anxiety, psychological distress) or alcohol use scales.

##### 3.1.4.1 Primary outcomes

Primary outcome measures of effectiveness include reduction of:

Depressive symptoms
Alcohol use

All outcomes must be assessed with validated mental health scales.

##### 3.1.4.2 Secondary outcomes

We will also include the following secondary outcomes when reported in eligible studies, but these will not be used to determine eligibility for inclusion in the review:

Psychological distress symptoms
Anxiety symptoms

##### 3.1.4.3 Duration of follow‐up

All durations of follow‐up will be eligible for inclusion. We will categorize follow‐up as short, medium or long term based on previous literature as follows:

- Short term: 3 months or less
- Medium term: Between 6 and 12 months follow‐up
- Long term: Longer than 12 months follow up

##### 3.1.4.4 Types of settings

Mobile apps for depressive symptoms or alcohol use delivered in any setting will be eligible for inclusion.

### 3.2 Search methods for identification of studies

A search strategy will be developed and peer‐reviewed by a librarian with expertise in systematic review searching.

#### 3.2.1 Electronic searches

We will search the following bibliographic databases: MEDLINE (via Ovid), Embase (via Ovid), PsycINFO (via Ovid), CINAHL (via EBSCO Host), and CENTRAL (via the Cochrane Library). We will also search ClinicalTrials.gov and the World Health Organization (WHO) International Clinical Trials Registry Platform (ICTRP) to identify ongoing or recently completed studies. The search will be restricted from January 1, 2008 to July 2021 to coincide with the release of the most popular online software stores and app distribution services such as Google Play (formerly known as the Android Market) (Android Developer's Blog, 2008) and Apple's App Store (Apple, 2008). There will be no language restrictions. The search will use a combination of indexed terms, free text words, and MeSH headings. See Table 1 for sample search strategy.

**TABLE 1:** Sample search strategy

Sample search strategy (Medline)
1 (teen* or youth* or adolescen* or juvenile* or (young adj2 (male or males or female* or adult* or person* or individual* or people* or population* or man or men or wom#n)) or youngster* or highschool* or college* or ((secondary or high* or univ*) adj2 (school* or education or student*))).ti,ab,kf. or adolescent/ or young adult/
2 Mental Health/ or Depression/or Anxiety/ or Gambling/ or Self‐Injurious Behavior/ or "Sleep Initiation and Maintenance Disorders"/ or exp Mental Disorders/ or exp suicide/ or suicidal ideation/ or suicide, attempted/ or exp Sleep Wake Disorders/ or exp Substance‐Related Disorders/
3 (mental health* or wellbeing or well‐being or anxiety* or anxious or depress* or ptsd or posttrauma* or post‐trauma* or suicid* or bipolar* or psycho* or (sleep* adj2 (disorder* or syndrome* or problem*)) or traumatic stress* or insomnia* or parasomnia* or dyssomnia* or adhd).ti,ab,kf.
4 ((attention or hyperactiv*) adj3 (deficit or disorder)).ti,ab,kf.
5 ((substance* or opioid* or marijuana or cannabis or cannabinoid* or alcohol) adj3 (abus* or misus* or disorder* or overuse*)).ti,ab,kf.
6 ((eating or feed* or food*) adj2 disorder*).ti,ab,kf.
7 (self adj2 (harm* or injur* or mutilat*)).ti,ab,kf.
8 2 or 3 or 4 or 5 or 6 or 7
9 Mobile applications/ or Cell Phone/
10 (Mhealth* or m‐health* or app or apps or mobile or portable or (cell adj2 phone) or smartphone).ti,ab,kf.
11 9 or 10
12 Randomized controlled trials as Topic/ or Randomized controlled trial/ or Random allocation/ or Double blind method/ or Single blind method/ or Clinical trial/ or exp Clinical Trials as Topic/
13 ((clinic$ adj trial$1) or ((singl$ or doubl$ or tripl$) adj (blind$3 or mask$3)) or randomly allocated or placebo* or (allocated adj2 random) or randomized controlled trial or randomi?ed or controlled clinical trial).tw.
14 12 or 13
15 1 and 8 and 11 and 14
16 Animals/ not Humans/
17 15 not 16
18 limit 17 to yr=“2008 ‐Current”

### 3.3 Data collection and analysis

#### 3.3.1 Description of methods used in primary research

Eligible publications will always report the findings of a randomized trial.

##### 3.3.1.1 Example: Mobiletype

A mobile app called Mobiletype was developed and its feasibility was tested in a school‐based study and clinical study among adolescents who consume alcohol (Kauer et al., 2009). Participants answered questions about their daily activities, alcohol use, stressors, and negative mood four times a day for 1 week. Recommendations for future studies were proposed, and Mobiletype was then evaluated using a randomized controlled trial with adolescents who reported elevated levels of distress (Kauer et al., 2012, Reid et al., 2011, 2013). Patients aged 14–24 years were recruited from rural and metropolitan general practices. Healthcare providers identified and referred eligible participants (those with mild or more mental health concerns) who were randomly assigned to either the intervention group (where mood, stress, and daily activities were monitored) or the attention comparison group (where only daily activities were monitored). Participants completed pre‐, post‐, and 6‐week posttest measures.

#### 3.3.2 Selection of studies

Two review authors will independently assess all records yielded by our search against our eligibility criteria. A two‐part study selection process will be used; the first step will include screening titles and abstracts of all records yielded by our search against our eligibility criteria, the second step will include screening the full text of included records. We will report published trial protocols in our PRISMA search and screening flowchart. Before initiating the screening process, two reviewers will undertake a screening exercise with a random sample of *n* = 100 records to ensure inter‐reviewer agreement and calibrate the screening strategy as necessary. Inter‐rater agreement will be measured using the *κ* coefficient (Cohen, 1960), and a *κ* statistic of 0.81 or higher will be deemed an indication of adequate inter‐reviewer screening reliability (Landis & Koch, 1977). We will resolve any disagreements through discussion or, if required, we will consult a third review author.

#### 3.3.3 Data extraction and management

##### 3.3.3.1 Details of coding categories

We will develop a standardized data extraction sheet. This extraction framework will be piloted with a random sample of *n* = 10 included records and revised accordingly to ensure the validity of the data extraction form. This iterative process will ensure that our data extraction process is compatible with our analysis objectives. Two reviewers will extract data in duplicate and independently and compare results afterwards. Any discrepancies in data extraction will be resolved by discussion or with the help of a third reviewer.

Reviewers will extract the following variables: (1) Context of the study: geographical, epidemiological, socioeconomic, socio‐cultural, political, legal, and ethical contextual data; (2) Study methodology: objective, study design, methodological details such as processes for randomization, allocation and blinding, target population, recruitment and sampling procedures, setting, participant eligibility criteria, and participant baseline characteristics; (3) Intervention: name, description, components (e.g., timing, frequency, route of delivery), and details of the comparison intervention; (4) Outcomes: Definitions, instrument and scale interpretation, timing of outcome measures, and adverse events; (5) Results: Participant follow up, binary (dichotomous) data, continuous data, between‐group estimates, and qualitative key findings; and (6) Author conclusions, funding and conflict of interest. Throughout data extraction we will pay special attention to participant characteristics and/or outcomes stratified by PROGRESS‐Plus: Place of residence, Race/ethinicity, Occupation, Gender/sex, Religion, Education, Socioeconomic status, Social capital and other factors associated with unequal opportunities for good health such as age, disability and sexual orientation (Attwood et al., 2016; Welch, 2013).

#### 3.3.4 Assessment of risk of bias in included studies

We will use the Cochrane risk of bias tool (Higgins et al., 2011) to appraise the quality of the methods used in the parent randomized trial. This tool will allow us to report on any potential selection, performance, or detection biases that may affect our internal and external validity and over‐ or under‐estimate the true intervention effect. Two reviewers with expertise in epidemiological methods and bias assessment will be designated to undertake the critical appraisal assessment independently. The reviewers will provide their judgments (low‐risk, high‐risk, or unclear risk) on each category of the risk of bias tool alongside a justification that supports their judgment. Categories of interest to be evaluated are: Randomization, allocation concealment, blinding of participants and personnel, blinding of outcome assessment, incomplete outcome data, and selective outcome reporting.

#### 3.3.5 Measures of treatment effect

We will categorize effectiveness data based on intervention purpose and select data based on our specified outcomes. Outcome data measured using categorical scales will be synthesized using relative risk measures (i.e., relative risks or odds ratios) or risk difference measures (absolute risks or attributable risks). If given the choice, we will report relative risk (RR) instead of odds ratios (OR) because the latter tend to overestimate effect estimates in prospective experiments (Knol et al., 2012). Outcome data measured using continuous scales will be synthesized as the mean difference or the mean difference from baseline to reflect the intergroup change in effect estimates over time. All effect estimates will be accompanied by measures of variance (standard deviations) and statistical significance estimates (confidence intervals or *p* values) when possible at the *a* = 0.05 level of significance, unless stated otherwise. We will aim to calculate measures of variance and statistical significance whenever the authors do not provide them (Borenstein, 2009) We will calculate NNT from all statistically significant effects for categorical outcomes.

#### 3.3.6 Unit of analysis issues

We will assess the unit of analysis of all the trials to individuals. Our synthesis will include individual effect sizes using the robust standard error approach to handle multiple effect sizes when appropriate (see data synthesis). Where possible, effects sizes will be computed for our selected outcomes within each study. In the event that a study provides more than one effect size for a particular outcome, our approach will be to drop outcomes. This will involve selecting the outcome that is most similar to those used by other studies in that category and retaining only that particular effect size in the analysis. In cases where a single evaluation of effectiveness provides data on multiple outcome measurements (i.e., depression and anxiety measurements) we will also report those findings based on outcome measurement to ensure that the effectiveness of the intervention at hand is depicted clearly by each of our outcomes of interest.

#### 3.3.7 Dealing with missing data

We will contact study authors to obtain missing data relevant from standard deviation when necessary. If we fail to get data, effect estimates will not be included in the pooled meta analyses, but we will report narratively as reported by authors. We will choose unadjusted means when available and use the intent‐to‐treat data when available.

#### 3.3.8 Assessment of heterogeneity

To facilitate the process of assessing for clinical heterogeneity between studies, we will standardize the PICO definitions (population, intervention, comparison, and outcome) of each included study with definitions that align with the scope of our review (McKenzie, 2019). One reviewer will write the full text publication and/or data extraction form for each included study and make a decision about the category in which each component of the study PICO falls. A second reviewer will verify these decisions and any discrepancies will be resolved by reaching a consensus or consulting a third reviewer. The categories we will use to standardize the PICO definitions are presented below.

**Table:** 

PICO	PICO definitions	Standardized PICO categories
Population	Youth selected to have the condition	Youth with mental health condition
	Youth had the condition at baseline	
Intervention	Smartphone application to manage an existing or assumed to exist condition	Management‐based intervention
Comparison	Sham intervention	Controlled intervention
	Placebo intervention	
	No intervention	Withheld comparison
	Waitlisting	
	Usual/standard care	
Outcome	Depression symptoms	Common mental health symptoms
	Anxiety symptoms	
	Psychological stress	
	Alcohol use	Substance use

Studies will, first, be grouped, to recognize the inherited differences in the design and therapeutic dose of the active treatment component between management‐based interventions. Determining the intervention purpose requires reviewing the rationale and theory behind the intervention development and implementation. Secondly, studies that measured the same outcome (e.g., severity of depression symptoms) will be assessed for clinical heterogeneity by examining whether their standardized PICO definitions aligned with each other. The same structure (intervention purpose > outcome) will be used to synthesize and report results.

Furthermore, we will aim to assess the statistical heterogeneity of meta‐analyzed results by examining the *I* ^2^ and *χ* ^2^ estimates calculated using RevMan 5.3. We will estimate the percentage of the total variability due to heterogeneity using *I* ^2^ values; 0% representing no heterogeneity, 50% indicating moderate heterogeneity and 75% indicating high heterogeneity (Higgins et al., 2003). When, and if, we detect statistical heterogeneity, we will explore the clinical variability of studies that contributed to this heterogeneity. We will investigate heterogeneity when appropriate with subgroup analysis considering gender and socioeconomic status.

#### 3.3.9 Assessment of reporting biases

We will use the ROB 2.0 criteria related to selective outcome reporting to assess for reporting bias. We will report all protocols from search that did not have a published study. We will assess for publication bias with funnel plot with all meta analyses with *n* = 10 or more studies (Cumpston et al., 2019).

#### 3.3.10 Data synthesis

We will synthesize results from continuous outcomes as mean differences at follow‐up, whereas results from categorical outcomes will be synthesized as relative risk measures, such as odds ratios and risk ratios. Whenever possible, we will prioritize risk ratios over odds ratios because the latter tend to overestimate the effect size (Knol et al., 2012). All effect estimates will be accompanied by estimates of statistical significance, such as 95% confidence intervals and p values. We will set the threshold of statistical significance at the *a* = 0.05 level unless reported otherwise in the study from which the result will be synthesized.

Whenever clinical homogeneity allows, we will meta‐analyze results and create first plots using RevMan 5.4. We will use a random‐effects model that calculates a mean pooled result, working under the assumption that there is no one true effect estimate and that the effect estimate of each included study falls on a normal distribution of the effect estimate (Borenstein, 2009). We have chosen a random‐effects model to account for the inherited heterogeneity between the characteristics of study cohorts, intervention design, and implementation context. Results that are not pooled together will be synthesized narratively (Popay et al., 2006). We will tabulate all results and order them in descending fashion using GRADE certainty of evidence tables.

All results will be accompanied by a GRADE certainty assessment which considers the precision of the effect estimate (information size and width of confidence interval). If a study used any methodology to control for a certain covariate outside of the PROGRESS + criteria (O'Neill et al., 2014), we will report the adjusted or “corrected” effect estimates and point out the methods used.

#### 3.3.11 Subgroup analysis and investigation of heterogeneity

We will investigate any important heterogeneity using subgroup analysis considering gender, race/ethnicity socioeconomic status and considerations.

#### 3.3.12 Sensitivity analysis

We will conduct sensitivity analyses for outliers when necessary, exploring the clinical variability of studies that contributed to this heterogeneity.

#### 3.3.13 Summary of findings and assessment of the certainty of the evidence

We will assess the certainty of the evidence using the GRADE approach. Results will be presented using GRADE Evidence Profiles.

## CONTRIBUTIONS OF AUTHORS

- Content: Kevin Pottie, Dominique Ranger, Rinila Haridas, Olivia Magwood
- Systematic review methods: Kevin Pottie, Jennifer Petkovic, Olivia Magwood
- Statistical analysis: Ammar Saad, Shahab Sayfi
- Information retrieval: Olivia Magwood, Kate Volpini, Franklin Rukikamirera, Yvonne Tan, Jeremie Alexander

## DECLARATIONS OF INTEREST

The authors declare no potential conflicts of interest.

### 1 PRELIMINARY TIMEFRAME

Approximate date for submission of the systematic review: December 15, 2021

### 2 PLANS FOR UPDATING THIS REVIEW

Kevin Pottie will be responsible for updating this review every 5 years.
